# SNEDDS Containing Poorly Water Soluble Cinnarizine; Development and *in Vitro* Characterization of Dispersion, Digestion and Solubilization

**DOI:** 10.3390/pharmaceutics4040641

**Published:** 2012-12-14

**Authors:** Anne T. Larsen, Anayo Ogbonna, Ragheb Abu-Rmaileh, Bertil Abrahamsson, Jesper Østergaard, Anette Müllertz

**Affiliations:** 1 Department of Pharmacy, Faculty of Health and Medical Sciences, University of Copenhagen, Universitetsparken 2, DK-2100 Copenhagen, Denmark; E-Mails: anne.tungelund@sund.ku.dk (A.T.L.); annyogbonna@yahoo.com (A.O.); jesper.oestergaard@sund.ku.dk (J.Ø.); 2 AstraZeneca, Pharmaceutical Development, Formulation Science, Macclesfield, United Kingdom; E-Mail: ragheb.aburmaileh@astrazeneca.com; 3 AstraZeneca, Pharmaceutical Development, Physical Science, Mölndal, Sweden; E-Mail: bertil.abrahamsson@astrazeneca.com; 4 Bioneer: FARMA, Danish Drug Development Center, Copenhagen, Denmark

**Keywords:** self-nanoemulsifying drug delivery system, cinnarizine, cryo-TEM, dynamic light scattering, droplet size

## Abstract

Self-Nanoemulsifying Drug Delivery Systems (SNEDDSs) were developed using well-defined excipients with the objective of mimicking digested SNEDDSs without the use of enzymes and *in vitro* lipolysis models and thereby enabling studies of the morphology and size of nanoemulsions as well as digested nanoemulsions by Cryo-TEM imaging and Dynamic Light Scattering. Four SNEDDSs (I-IV) were developed. Going from SNEDDS I to IV lipid content and solubility of the model drug cinnarizine decreased, which was also the case for dispersion time and droplet size. Droplet size of all SNEDDS was evaluated at 1% (*w*/*w*) dispersion under different conditions. Cinnarizine incorporation increased the droplet size of SNEDDSs I and II whereas for SNEDDSs III and IV no difference was observed. At low pH cinnarizine had no effect on droplet size, probably due to increased aqueous solubility and partitioning into the aqueous phase. Dispersion of the SNEDDSs in Simulated Intestinal Media (SIM) containing bile salts and phospholipids resulted in a decrease in droplet size for all SNEDDS, as compared to dispersion in buffer. Increasing the bile salt/phospholipid content in the SIM decreased the droplet sizes further. Mimicked digested SNEDDS with highest lipid content (I and II) formed smaller nanoemulsion droplet sizes upon dispersion in SIM, whereas droplet size from III and IV were virtually unchanged by digestion. Increasing the bile acid/phosphatidylcholine content in the SIM generally decreased droplet size, due to the solubilizing power of the endogenous surfactants. Digestion of SNEDDSs II resulted in formation of vesicles or micelles in fasted and fed state SIM, respectively. The developed and characterized SNEDDS provide for a better knowledge of the colloid phases generated during digestion of SNEDDS and therefore will enable studies that may yield a more detailed understanding of SNEDDS performance.

## 1. Introduction

Lipid-based drug delivery systems enhance the bioavailability of many poorly water-soluble compounds, *i.e.*, compounds belonging to the Biopharmaceutical Classification System class II and IV [1,2]. Multiple mechanisms have been implicated in the bioavailability enhancing properties of lipid-based formulations. Lipid-based drug delivery systems can increase the rate and extent of *in vivo* solubilization. The drug is usually administered as a solution with excipients that facilitate solubilization of the drug during gastro-intestinal transit. Additionally, excipients used in lipid-based drug delivery systems can prolong the gastric emptying time and stimulate the release of endogenous surfactants such as bile salts and phospholipids [3]. Some excipients used in lipid-based drug delivery systems can inhibit intestinal efflux transporters [4]. All the above mentioned factors can increase intestinal absorption [5]. Excipients can also inhibit metabolizing enzymes, such as the cytochrome P450 enzymes, which may eventually lead to increased bioavailability [6]. Furthermore, lipidic excipients can facilitate lymphatic transport of compounds absorbed by this route [7]. An advantage of facilitating lymphatic transport is that the bioavailability increases due to reduced first pass metabolism, as this route bypasses the liver.

Especially the use of self-nanoemulsifying drug delivery systems (SNEDDSs) is of interest. These types of delivery systems are administered as a preconcentrate and disperse in the gastric and intestinal fluids to form nanoemulsions. The formed emulsions are transparent with an opalescent appearance. There is a certain amount of confusion as to the terminology and differences between SNEDDSs and self-microemulsifying drug delivery systems (SMEDDSs). Nanoemulsions are kinetically stable systems whereas microemulsions are thermodynamically stable [8,9,10,11]; Nanoemulsions will phase separate over time, whereas microemulsions are in their most favorable state and are therefore stable if chemical degradation of its components can be prevented. The systems developed as part of this work are SNEDDSs.

A SNEDDS is typically composed of four classes of excipients: lipids, surfactants, co-surfactants, and co-solvents in a ratio that will allow the formulation to form a nanoemulsion upon dispersion in aqueous media. The ability of the formed nanoemulsion to keep the drug compound in solution after dispersion and digestion has been accepted as a critical property for the *in vivo* performance of SNEDDSs [12]. However, the drug compound is in solution in the nanoemulsion oil droplets rather than in aqueous solution where it needs to be prior to absorption across the intestinal membrane. The processes taking place in the gastrointestinal tract after ingestion of SNEDDSs are complex and not fully understood. Previous studies have shown that the bioavailability of two poorly water soluble compounds decreased when administered in a SMEDDS or a SNEDDS to bile depleted rats compared with normal rats [13,14]. A better understanding of how SNEDDSs interact with the environment of the GI tract may be achieved using well-characterized SNEDDSs specifically designed for this purpose. The approach presented in the current study makes use of such well-characterized SNEDDSs, which may be relevant for future studies as it is difficult to employ samples obtained from *in vitro* lipolysis experiments in other *in vitro* characterization models. Challenges include controlling the degree of digestion and terminating the lipolysis process without significantly altering the system. 

**Figure 1:**
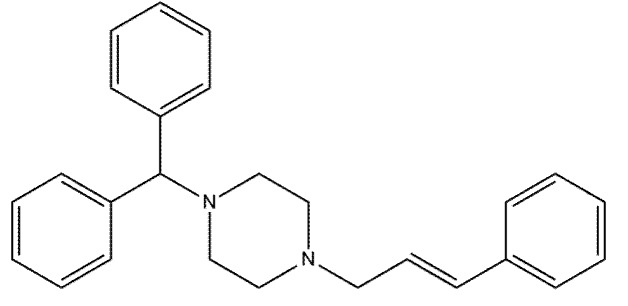
Chemical structure of cinnarizine.

The aim of the present work was to develop SNEDDSs composed of well-defined excipients, which can be utilized to study the colloidal structures generated during digestion, without the use of digestive enzymes. The impact of bile salts and phospholipids and different degrees of digestion, on the colloid structures could this way be studied. This approach enables studies of the morphology and size of these colloidal structures by Cryo-TEM imaging and Dynamic Light Scattering (DLS). The poorly water-soluble Biopharmaceutical Classification System class II compound cinnarizine [15] was used as model drug compound. Selected physiochemical properties are given in Table 1 and the chemical structure of cinnarizine is shown in Figure 1.

**Table 1:** Selected Physiochemical Properties for Cinnarizine.

Mw	368.5
Log P	5.77 [16]
pK_a_	7.47 [17]
	1.95 [18]
Aqueous solubility (37 °C) *^a^*	0.39 ± 0.1 µg/mL *^a^*

*^a^* Determined in the present study, TRIS maleate buffer pH 6.5 (*n* = 3).

## 2. Materials and Methods

### 2.1. Materials

Oleic acid (composition from certificate of analysis: 0.4% C16, 2.5% C18, 89.2% C18–1, 5.9% C18–2, 1.0% C18–3), Rylo MG19 Pharma (composition from certificate of analysis: 96% monoglyceride, 4% diglyceride, 0.1% free glycerine, fatty acid composition: 0.2% C16, 2.7% C18, 90% C18–1, 4.3% C18–2, 0.7% C18–3, 0% C20, 1.5% C20–1), and Rylo MG13 Pharma (composition from certificate of analysis: 98% monoglyceride, 1.5% diglyceride, 0.1% free glycerine, fatty acid composition: 7.4% C16, 3.6% C18, 21.8% C18–1, 65.3% C18–2, 0.3% C18–3, 0.3% C20, 0.3% C20–1) were all gifts from Danisco (Copenhagen, Denmark). Phosphatidylcholine SPC (≥98%) was purchased from Lipoid GmbH (Ludwigshafen, Germany). Cremophor RH40 was obtained from BASF (Ludwigshafen, Germany). Palmitic acid (≥99%), stearic acid (≥98.5%), Brij 97, cinnarizine (≥99%), TRIS maleate, and sodium taurocholate (≥95%) were purchased from Sigma-Aldrich (Saint Louis, MO, USA). Polyethylene glycol 400 (Ph. Eur. grade), sesame oil (Ph. Eur.), and linoleic acid (≥99%) were obtained from Fluka (Buchs, Switzerland). Ethanol (Ph. Eur. grade) and acetonitrile (HPLC grade) were purchased from VWR (Herlev, Denmark).

### 2.2. Development of Self-Nanoemulsifying Drug Delivery Systems

The excipients used were selected in such a way that when the SNEDDSs had been developed and identified it would be possible to prepare these SNEDDSs as lipolysed (thus mimicking the composition of digested) SNEDDSs without the use of lipases as described in Section 2.8. Preparation of Mimicked Digested SNEDDS. The rationale behind the selection of excipients is explained further in Section 3.1. Selection of Excipients: A series of mixtures with varying ratios of lipid, surfactant and co-solvent was prepared. System A: mixtures of Cremophor RH40, sesame oil, and oleic acid. System B: mixtures of Cremophor RH40, sesame oil, and Brij 97. System C: mixtures of Cremophor RH40, sesame oil + oleic acid (4:3 (*w*/*w*)), and Brij 97. System D: mixtures of Cremophor RH40, sesame oil + oleic acid (4:3 (*w*/*w*)), and Brij 97 + PEG400 (2:1 (*w*/*w*)). The ratio of sesame oil to oleic acid was selected from preliminary studies and a maximum of 20% Brij 97 and 10% PEG400 was applied due to considerations on toxicity and prevention of precipitation, respectively. The ratios investigated are shown in Table 2.

**Table 2:** The ratios of different excipients examined in the present study (% *w*/*w*).

Excipient	System A	System B	System C	System D
Sesame oil	10–80	10–80	5.7–45.7	5.7–45.7
Oleic acid	10–80	-	4.3–34.3	4.3–34.3
Cremophor RH 40	10–80	10–80	10–80	10–80
Brij 97	-	10–20	10–20	7.7–20
PEG 400	-	-	-	3.3–10
Ethanol	0, 10	0, 10	0, 10	0, 10

The various components were weighed in glass vials and mixtures were heated to 50 °C until all components were melted. Hereafter, mixing by means of end-over-end rotation overnight at ambient temperature (approx. 22–25 °C) while protected from light was applied. The mixtures were visually inspected for homogeneity. After evaluation of the mixtures, ethanol was added to a final concentration of 10% (*w*/*w*). The ethanol containing samples were subjected to end-over-end rotation overnight at ambient temperature, protected from light. The homogeneous mixtures were tested for their nanoemulsion forming properties by visual inspection. A 1 g mixture was added to a beaker containing 100 mL of water at 37 °C, and gentle stirring was applied. A dispersion that formed a mono-phasic clear and/or slightly opalescent system within 10 min was considered a suitable SNEDDS.

### 2.3. Solubility of Cinnarizine in SNEDDSs, Excipients and Buffer

An excess of cinnarizine was added to the SNEDDSs, selected excipients (sesame oil and oleic acid) and 100 mM TRIS maleate buffer, pH 6.5. The resulting suspensions were placed in an end-over-end rotating device protected from light at 25 °C for the SNEDDSs and at 37 °C for the excipients and buffer; the temperatures were selected for storage purpose and for its physiological relevance, respectively. Samples were withdrawn at specified time points, *i.e.*, 28, 50, and 72 h for the SNEDDSs, 22 h and 48 h for the excipients, and at 51 h and 75 h for the buffer. Samples were centrifuged at 15,000 rpm for 45 min (Biofuge 15, Hereaus-Sepatech, Oterode, Germany). Between two and three drops of supernatant were weighed in a 10 mL volumetric flask and diluted with ethanol. This solution was further diluted with mobile phase to an appropriate concentration. Samples from the buffer were diluted with mobile phase. Quantification of cinnarizine was performed as described in the analytical section. The determination of solubility was considered attained when samples from two consecutive time points varied by less than 5%. The solubilities were determined in triplicate.

### 2.4. Quantitative Analysis of Cinnarizine

The concentration of cinnarizine was determined using a HPLC method. A HPLC system (Hitachi, Merck, Darmstadt, Germany) equipped with a D-7000 interface, an L-7200 autosampler, a L-7100 pump, a L-7300 column oven (35 °C), and a L-7400 UV detector or a L-7480 fluorescence detector. Data was processed by using HPLC System Manager Version 4.0 from Merck (Darmstadt, Germany). Chromatographic separation was obtained using a Luna C18 150 × 4.6 mm, 5 µm, column and a C18 guard column (Phenomenex, Torrance, CA, USA). The mobile phase consisted of 50% (*v*/*v*) acetonitrile and 50% (*v*/*v*) 20 mM ammonium dihydrogen phosphate monobasic buffer, pH 4.5. The temperature of the column was maintained at 35 °C and the flow rate and injection volume were 1 mL/min and 25 µL, respectively. The UV detection was carried out at 253 nm and quantification was performed from peak areas using a six point linear standard curve ranging from 0.25–4 µg/mL. In the case of fluorescence detection (solubility in buffer) excitation at 249 nm and emission at 311 nm were used and quantification was performed from peak areas using a six point linear standard curve ranging from 5–160 ng/mL.

### 2.5. Colloidal Dispersion of SNEDDSs

Colloidal dispersion studies were performed in 250 mL fasted-state, simulated gastric fluid (FaSSGF) at pH 1.6. The FaSSGF was prepared as described by Vertzoni *et al.* [19] and contained 80 µM sodium taurocholate, 20 µM phosphatidylcholine, 0.1 mg/mL pepsin and 34.2 µM sodium chloride. The temperature was 37 °C and a DT70 USP II dissolution apparatus from ERWEKA (Heusenstamm, Germany) was used. The paddles were mounted right below the surface of the dispersion medium, and an agitation speed of 100 rpm was applied. A quantity of 1 g of each SNEDDS containing 25 mg cinnarizine was weighed on a watch glass, and the glass with formulation was gently slid down the vessel wall to the bottom of the dissolution vessel and thereby introduced into the dispersion medium. The dispersion time was defined as the time it took for the formulation residue to disappear. Samples of 1000 µL were taken at 1, 2.5, 5, 10, 15, 20, 30, 40, 50, and 60 min upon addition of the formulation. Samples were centrifuged (Biofuge 13, Hereaus-Sepatech, Oterode, Germany) at 6366*g* for 10 min. Samples of the supernatant were diluted appropriately with mobile phase and were assayed for cinnarizine content by HPLC as described in the analytical section. The content of the supernatant represents the cinnarizine content, which has not precipitated (cinnarizine in solution + cinnarizine in the nanoemulsion droplets).

### 2.6. Analysis of Triglyceride Composition in Sesame Oil

Quantitation of triglycerides in the sesame oil was performed, with minor modifications according to the European Pharmacopoeia 6.5 monograph: “Sesame Oil Refined”. Liquid chromatography with evaporative light scattering detection (ELS) was performed using a HPLC system (Hitachi, Merck, Darmstadt, Germany) equipped with a D-7000 interface, a L-7200 autosampler, a L-7100 pump, a L-7300 column oven (45 °C), and an ELS detector (PL-ELS 2100, Polymer Laboratories, Church Stretton, UK) with an evaporator temperature of 85 °C, nebuliser temperature of 45 °C and nitrogen carrier gas flow of 2 mL/min. The separation was achieved by coupling two Luna C18 (5 µm, 250 mm × 4.6 mm) columns from Phenomenex (Torrance, CA, USA). Gradient elution was applied. Mobile phase A: Acetone, methylene chloride, acetonitrile (5:15:80 *v*/*v*/*v*). Mobile phase B: Acetone, methylene chloride, acetonitrile (20:20:60 *v*/*v*/*v*). The flow rate was 0.7 mL/min and the following gradient was used: 0–21.5 min 100% A → 75% A, 21.5–36 min 75% A → 75% A, 36–100 min 75% A → 0% A, 100–107.5 min 0% A → 100% A, 107.5–120 min 100% A → 100% A. Samples and standards were diluted in acetone and methylene chloride (50:50 *v*/*v*). Triolein was used as a standard (0.02–1.6 mg/mL). The standard curve was obtained by plotting the logarithm of the obtained area against the logarithm of the triolein concentration. The correlation coefficient (*r*^2^) of the standard curve was 0.999. The triglyceride composition was determined in two different sesame oil batches, 1354884 53207P01 and 1367639 14507P07, respectively.

### 2.7. Preparation of Simulated Intestinal Media

Two simulated intestinal media (SIM) were prepared, SIM_low_ and SIM^high^ simulating a fasted and fed state, respectively. The pH of both media was 6.5 and the composition is shown in Table 3. Sodium chloride was added to obtain a 270 mOsm solution and NaN_3_ was added to prevent bacterial growth. SIM_low_ and SIM^high^ contained sodium taurocholate and phosphatidylcholine 5:1.25 mM and 15:3.75 mM, respectively. The two intestinal simulated media were prepared as previously described [20].

**Table 3:** Composition of SIM.

	SIM_low_	SIM^high^
Sodium taurocholate (mM)	5	15
Phosphatidyl choline (mM)	1.25	3.75
NaCl (mM)	77	65
Trizma maleate (mM)	100	100
NaN_3_ (mM)	3	3
Osmolarity (mOsm)	270	270
pH	6.5	6.5

### 2.8. Preparation of Mimicked Digested SNEDDSs

Mimicked digested SNEDDSs were prepared by replacing sesame oil with fatty acids and monoglycerides. In the calculation of the composition of fatty acids resulting from digestion of sesame oil, hydrolysis of the ester bonds at position 1 and 3 was assumed. The triglyceride composition determined as described above was utilized to calculate the amount of different fatty acids and monoglycerides that had to be mixed to mimic sesame oil digestion.

A mimicked 100% digested SNEDDS formulation was prepared by weighing Cremophor RH40, oleic acid, Brij 97, PEG 400, and ethanol in glass vials in the same ratio as when intact SNEDDSs were prepared (Table 4). The sesame oil was replaced by a fatty acids/monoglyceride mixture (2:1 ratio) with a composition as follows: 278 mg oleic acid, 267 mg linoleic acid, 21 mg stearic acid, 74 mg palmitic acid, 207 mg monooleic glyceride, and 248 mg monolinoleic glyceride. The mimicked digested formulation was mixed overnight. For preparation of partly digested SNEDDSs, a mimicked 100% digested SNEDDS was mixed with an appropriate amount of intact SNEDDS. The oleic acid and monooleic glyceride preparations applied were not pure with regard to fatty acid chain length, and this was taken into account in the calculations above.

**Table 4:** Composition of SNEDDSs (*w*/*w* %).

Excipient	SNEDDS I	SNEDDS II	SNEDDS III	SNEDDS IV
Sesame oil	27	20.6	10.3	5.1
Oleic acid	18	15.4	7.7	3.9
Cremophor RH 40	45	45	45	54
Brij 97		9	18	18
PEG 400			9	9
Ethanol	10	10	10	10

### 2.9. Particle Size Measurements by Dynamic Light Scattering

Particle size measurements using Dynamic Light Scattering were performed on a Zetasizer ZS ZEN 3600 (Malvern Instruments Ltd., Malvern, United Kingdom). Samples were prepared in disposable polypropylene test tubes. Aliquots of preconcentrate were weighed in the tube and dispersing medium was added by weighing of appropriate amounts to create the dispersions. Dilutions were 1% *w*/*w* unless otherwise stated. Samples were kept in a heating cabinet at 37 °C under end-over-end mixing for 2 h before measurements were conducted at 37 °C. Samples were prepared in triplicates and two to four measurements were performed on each. Size distributions were calculated by volume using Dispersion Technology Software from Malvern Instruments Ltd.

### 2.10. Cryo-TEM

The SNEDDSs or mimicked digested SNEDDSs were dispersed (1% *w*/*w*) in the different media prior to vitrification. The samples for the Cryo-TEM studies were prepared in a controlled environment vitrification system (CEVS). A small amount of the sample (5 μL) was put on a carbon film supported by a copper grid and blotted with filter paper to obtain a thin liquid film on the grid. The grid was quenched in liquid ethane at −180 °C and transferred to liquid nitrogen (−196 °C). The samples were characterized with a TEM microscope (Philips CM120 BioTWIN Cryo, Eindhoven, The Netherlands) equipped with a post column energy filter (GATAN GIF 100, Pleasanton, CA, USA) using an Oxford CT3500 cryoholder and its workstation. The acceleration voltage was 120 kV and the working temperature was −180 °C. The images were recorded with a CCD camera (Gatan 791) under low dose conditions. The defocus was approximately 1 μm. Eight regions on each grid were identified and pictures were obtained from these different regions from each sample. Between 7 and 37 pictures were taken of each sample and a total of 292 pictures were obtained for this study.

## 3. Results and Discussion

Cinnarizine was used as a model drug compound in the present study. Cinnarizine is an interesting model drug to use in SNEDDSs due to its relatively high lipid solubility (Table 5) and a low and variable bioavailability. The bioavailability of cinnarizine from a conventional capsule and from an aqueous suspension formulation has been determined previously to be 0.8% ± 0.4% and 8% ± 4% in dogs (*n* = 4, mean ± SEM), respectively [21].

**Table 5:** Cinnarizine solubility in different excipients.

SNEDDS/Excipient	Solubility mg/g
SNEDDS I *^a^*	67.4 ± 0.9
SNEDDS II *^a^*	58.8 ± 0.5
SNEDDS III *^a^*	36.4 ± 0.7
SNEDDS IV *^a^*	28.3 ± 0.8
Sesame oil *^b^*	25.7 ± 0.4
Oleic acid *^b^*	335 ± 3

Mean ± SD (*n* = 3); *^a^* Determined at 25 °C; *^b^* Determined at 37 °C.

### 3.1. Selection of Excipients

The selection of excipients was motivated by the need to develop SNEDDSs with well-defined compositions. Therefore, only excipients with compositions were selected wherein the components could be analyzed, and/or where lipolysis in the gastro-intestinal tract can be considered to be minor.

Sesame oil was selected as the main lipid component of the SNEDDSs. Sesame oil is a vegetable oil used in food and can also be purchased as a Ph. Eur. grade product.

Oleic acid was selected because cinnarizine has a high solubility in oleic acid [22]. The properties of oleic acid vary with pH. The pK_a_ value of oleic acid in water is 9.85 [23], but the apparent pK_a_ shifts to lower values when oleic fatty acid is incorporated into mixed bile acid/phospholipid micelles (pK_a_ 6.5) [24]. The properties of ionized and protonated oleic acid are very different. Above the pK_a_, oleic acid acts like a conventional ionic hydrophobic surfactant whereas below the pK_a_, the properties of oleic acid resemble oil and oil droplets are formed when oleic acid is mixed with water [25]. For this reason, oleic acid is believed to behave like oil in the SNEDDSs at low pH (e.g., fasted stomach) and as a co-surfactant at neutral pH (e.g., fed stomach and fasted/fed intestine).

Cremophor RH40 was selected as the main hydrophilic surfactant. It has previously been used in SMEDDS formulations e.g., the marketed Sandimmune Neoral and Kaletra [26]. The components present in Cremophor RH40 include amphiphilic mono-, di- and tri-fatty acid (hydroxystearic acid) esters of polyethoxylated glycerol and polyethylenoxide, and in addition non-fatty acids esterified materials including glycerol polyoxyethylene ether and free polyethylene glycol [27]. Cremophor RH40 has a HLB between 14 and 16, and the CMC is reported to be 0.039% *w*/*v* [28]. *In vitro* lipolysis studies have shown that Cremophor RH40 is only hydrolysed to a minor extent (7.5%) [27]. In contrast, other surfactants are substrates for lipases *in vitro* e.g., Cremophor EL [27] and Labrafil M2125CS [29]. The use of hydrolysable surfactants results in the generation of a very complex mixture of lipolysis products during lipolysis. Such complex mixtures are difficult to mimick in mechanistic studies without the use of enzymes. Due to the limited lipolysis of the surfactant mixture in Cremophor RH40, it is expected to pass the gastrointestinal tract basically unaltered, resulting in a less complicated system.

Brij 97 was selected as an add-on surfactant. Brij 97 is a polyethylene monooleyl glycol ether and belongs to the group of polyoxyethylene alkyl ethers which are non-ionic surfactants [28]. Brij 97 is called an add-on surfactant here since it is in fact not a co-surfactant, because by definition a co-surfactant is a surface active agent that lowers the interfacial energy, but it cannot form micellar aggregates by itself [30]. Brij 97 is a hydrophilic surfactant with a HLB of 12.4 [28] and a CMC of 0.22 mM [31]. Brij 97 is a non-digestible surfactant [27] and is therefore expected to pass the gastrointestinal tract unaltered.

As hydrophilic co-solvents, PEG400 and ethanol were included in the formulations. Both can increase the solubility of the drug compound in the preconcentrate. They were included to aid the dispersion of the preconcentrate in an aqueous environment [32]. Furthermore, ethanol was used to obtain a homogeneous preconcentrate. A maximum of 10% of each co-solvent was used, since it is well known that co-solvents lose their solubilization capacity after dispersion of the system because they dilute into the aqueous continuum [32]. As a result, the drug compound may precipitate. 

### 3.2. Miscibility of Excipients and Identification of Nanoemulsifying Mixtures

**Figure 2:**
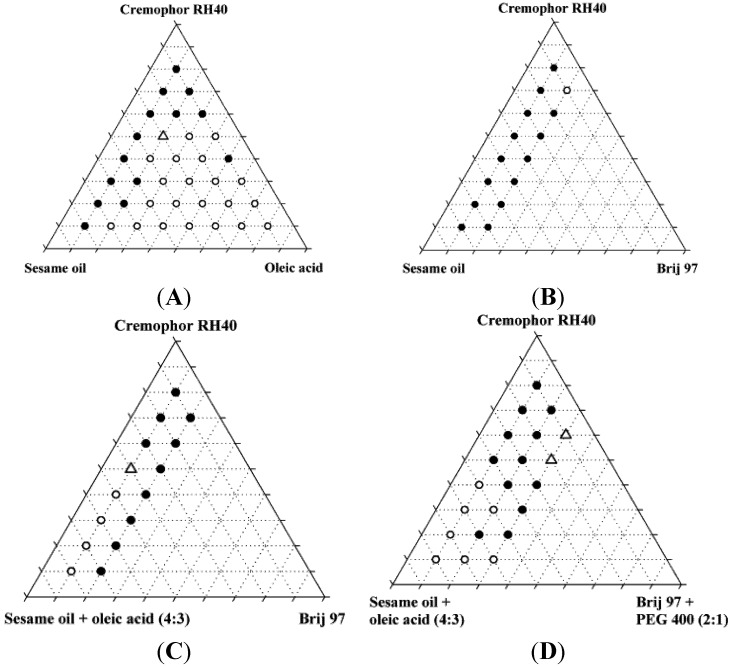
Miscibility of SNEDDS excipients depicted in pseudo-ternary diagrams. Additionally, these mixtures all contained 10% *w*/*w* ethanol. Solid circles are inhomogeneous mixtures, open circles are homogeneous mixtures, open triangles are homogeneous mixtures that forms nanoemulsions in water at a concentration of 1% (*w*/*w*).

The miscibility of the excipients in the four systems was investigated and additionally, the miscibility of the systems was investigated after addition of 10% (*w*/*w*) ethanol (Figure 2). A maximum content of 20% Brij 97 and 10% PEG400 was selected due to toxicity considerations and the risk of precipitation, respectively.

The miscibility of the excipients was poor in the absence of ethanol. Only two of the original 87 mixtures were homogeneous, and neither of these two mixtures was found to form nanoemulsions upon dispersion; instead, they formed coarse emulsions with a milky appearance. Upon addition of 10% ethanol, the number of homogeneous mixtures increased to 37 out of 87 of which four were found to form nanoemulsions when diluted with water. Ethanol is generally a good solvent for lipids and in the present study, it was able to increase the solubility of the lipids and surfactants in each other. Ternary diagrams where the points represent the mixtures investigated, are shown in Figure 2.

The reason for the relatively low number of homogeneous mixtures forming nanoemulsions was probably that co-surfactants e.g., 2-monoacylglycerides [26] were not used in the development of these SNEDDSs. The use of a co-surfactant can reduce the surface tension and reduce the bending stress of the interface, which aids the formation of nanoemulsions [33,34]. Furthermore, the area of nanoemulsion forming mixtures of oil, surfactant and co-surfactant in triangular diagrams is smaller when long chain triglycerides are used as compared with medium chain triglycerides [35,36].

The compositions of the four homogeneous mixtures (SNEDDSs) that were able to form nanoemulsions are shown in Table 4. Hereafter these systems are referred to as SNEDDS I, SNEDDS II, SNEDDS III, and SNEDDS IV. None of these four SNEDDSs fit into the Lipid Formulation Classification System (LFCS) put forward by Pouton 2006 [12], either they have too high a content of hydrophilic surfactant or too high a content of oil to fit into the various classes. Problems with assigning lipid-based formulations into the LFCS have been reported previously [26] and this emphasizes the complexity of lipid-based drug delivery systems. 

### 3.3. Solubility of Cinnarizine in SNEDDSs, Excipients and Buffer

The solubilities of cinnarizine in SNEDDS I-IV, sesame oil and oleic acid are shown in Table 5. The solubility of cinnarizine in 100 mM TRIS maleate buffer was 0.39 ± 0.1 µg/mL (*n* = 3). The solubility of cinnarizine in oleic acid obtained in this study (335 ± 3 mg/g at 37 °C) was somewhat higher than the solubility reported in a previous study (237 mg/g at 25 °C) [22]. The difference in temperature between these two measurements may cause the observed difference. Additionally a probable explanation is that oleic acid is a product of natural origin and therefore variations in composition between different suppliers may be expected. The oleic acid used in this study is 90% pure whereas the European Pharmacopeia specifies an oleic acid content between 65% to 88%.

**Figure 3:**
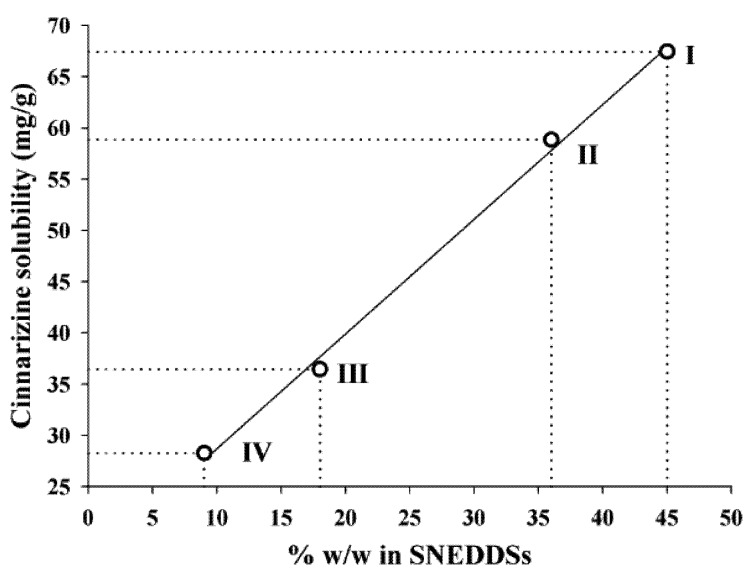
Relation between the cinnarizine solubility in SNEDDS at 25 °C and the total amount (% *w*/*w*) of lipid (sesame oil + oleic acid content), *r*^2^ = 0.997.

The solubility of cinnarizine in the SNEDDSs was linearly dependent on the total lipid content in the SNEDDSs (Figure 3). The correlation coefficients (*r*^2^) of the relation between solubility and amount of oleic acid + sesame oil were 0.997. Sesame oil and oleic acid are the most lipophilic excipients in the four SNEDDSs. Due to the lipophilic nature of cinnarizine (log P_Oct/W_ 5.77 [16]), these two excipients are important solvents of cinnarizine. However, due to the high solubility of cinnarizine in oleic acid this is probably the excipient that has the most significant impact on cinnarizine solubility in the SNEDDSs. The lipids are not expected to loose their capacity to keep cinnarizine in solution upon dispersion of the SNEDDSs because they are insoluble in water.

### 3.4. Colloidal Dispersion of SNEDDSs

**Figure 4:**
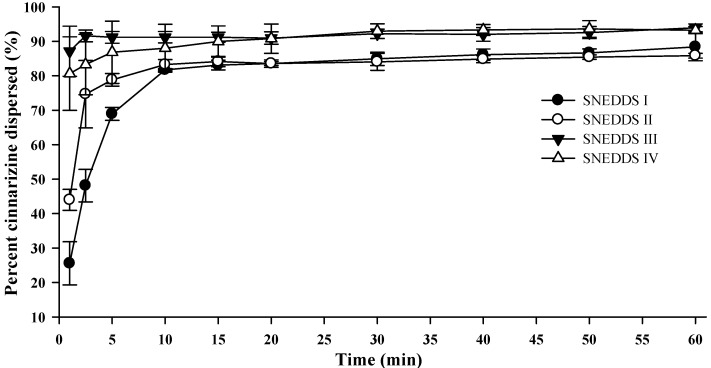
Dispersion of cinnarizine during dispersion of 1 g SNEDDS containing 25 mg cinnarizine per gram in fasted state simulated gastric fluids at pH 1.6 and 37 °C (*n* = 3).

Upon colloidal dispersion of SNEDDSs in FaSSGF (pH 1.6), cinnarizine was dispersed quickly and efficiently (Figure 4). Eighty percent of the dose was dispersed within 10 min for all four SNEDDSs. Precipitation of cinnarizine was not observed during the experiment (60 min) as was expected due to the high solubility of cinnarizine in water at low pH (853 µg/mL, pH 1.3 [37]). The release of cinnarizine was fastest from SNEDDSs III and IV and followed by SNEDDSs II and I, in that order. The dispersion time, defined as the time where formulation was no longer visible in the dissolution vessel, was 9.7 ± 1.5, 10.3 ± 2.0, 1.7 ± 0.6, and 1.8 ± 0.4 min (average ± S.D., *n* = 3) for SNEDDS I, II, III and IV, respectively. The faster dispersion of SNEDDS III and IV can be attributed to the higher content of hydrophilic surfactants and/or the addition of the co-solvent PEG 400. The dispersion time will be a function of the ease of emulsification, which is affected by how well water can penetrate the surface of the SNEDDS [36].

### 3.5. Droplet Sizes of SNEDDSs

The droplet size of the four SNEDDSs dispersed at 2%, 1% and 0.4% (*w*/*w*), in 100 mM TRIS maleate buffer (pH 6.5) was measured by DLS. Data confirmed that nanoemulsions were formed in the dispersion range investigated and that the developed systems were SNEDDSs since the droplet size was unaffected by the level of dispersion (data not shown). Distinguishing between SNEDDSs and SMEDDSs is controversial and subject to debate in the literature [38]. However, two tests can be performed to distinguish between nano- and microemulsions. First of all, the degree of dispersion of nanoemulsions does not impact droplet size and droplet distribution, whereas microemulsions change droplet size distribution with dispersion degree, and destruction of the one phase system can be observed [8]. Secondly the formation of nanoemulsions is dependent on the order of mixing of the components, surfactants and oil have to be premixed before mixing with water [8]. Conversely, the order of mixing does not alter the outcome of the system for microemulsions. The droplet size of nanoemulsions and microemulsions lies in the same range (10 to 300 nm [8,39] in diameter) and the size can therefore not be used to distinguish between the two.

**Figure 5:**
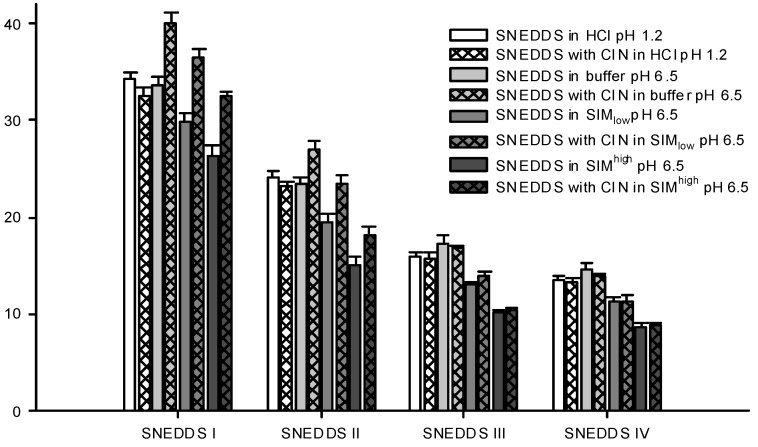
Particle sizes of formed nanoemulsions in various media determined by DLS. SNEDDSs with cinnarizine (25 mg/g SNEDDS) and without cinnarizine were dispersed in the media, 1% (*w*/*w*) at 37 °C. The bars represent mean ± SD (*n* = 3), between two and four measurements obtained on each sample.

The droplet size distributions of the formed nanoemulsions differed between the four SNEDDSs. SNEDDS III and IV formed the smallest nanoemulsion droplets and their sizes were in the same range, whilst SNEDDS I formed the largest droplets (Figure 5). This difference in size between the different formulations was consistent across all dispersion media. Apparently, larger amounts of lipid in the formulation produced larger droplet sizes and when lipid was replaced by surfactant smaller droplet sizes were obtained. This is consistent with previous investigations where a decrease in droplet size was observed with an increase in the surfactant to lipid ratio [40,41,42]. Table 6 shows the PDI values for the droplet size measurements shown in Figure 5 and generally when SNEDDSs were dispersed in buffer and HCl the PDI values were low indicating a monomodal droplet distribution. A relation between the droplet sizes of the dispersed SNEDDSs and the cinnarizine dispersion (Figure 4) was evident. Cinnarizine disperses fastest from SNEDDSs III and IV and these also have the smallest droplet size. This is probably an effect of the higher content of surfactants in these two SNEDDSs, which facilitate fast emulsification and the formation of smaller droplet sizes.

**Table 6:** Obtained polydispersity indexes from the droplet size measurements shown in Figure 5.

Media	PDI (Polydispersity Index)			
	SNEDDS I	SNEDDS II	SNEDDS III	SNEDDS IV
SNEDDS in HCl pH 1.2	0.052 ± 0.006	0.088 ± 0.014	0.033 ± 0.006	0.030 ± 0.006
SNEDDS with cinnarizine in HCl pH 1.2	0.093 ± 0.012	0.039 ± 0.006	0.078 ± 0.008	0.071 ± 0.017
SNEDDS in buffer pH 6.5	0.054 ± 0.009	0.040 ± 0.012	0.043 ± 0.018	0.059 ± 0.024
SNEDDS with cinnarizine in buffer pH 6.5	0.056 ± 0.008	0.047 ± 0.008	0.025 ± 0.005	0.099 ± 0.061
SNEDDS in SIM_low_ pH 6.5	0.097 ± 0.018	0.139 ± 0.058	0.223 ± 0.089	0.175 ± 0.040
SNEDDS with cinnarizine in SIM_low_ pH 6.5	0.080 ± 0.022	0.118 ± 0.023	0.178 ± 0.034	0.144 ± 0.031
SNEDDS in SIM^high^ pH 6.5	0.147 ± 0.016	0.158 ± 0.006	0.140 ± 0.049	0.168 ± 0.015
SNEDDS with cinnarizine in SIM^high^ pH 6.5	0.113 ± 0.010	0.158 ± 0.007	0.225 ± 0.038	0.117 ± 0.032

Differences in droplet size were not observed between the measurements at pH 1.2 (0.1 M HCl) and 6.5 (100 mM buffer) despite oleic acid becoming partly ionized at pH 6.5. One explanation to this could be that there is no difference between ionized and non-ionized oleic acids distribution in the nanoemulsion droplets. This is in accordance with previous findings where oleic acid was associated with the surface of the emulsion droplets of a submicron-emulsion both in the ionized and un-ionized state [43].

Sizes were measured for the dispersed SNEDDSs both with and without cinnarizine (25 mg cinnarizine per gram SNEDDS). Adding cinnarizine to the SNEDDSs increased the droplet size of SNEDDS I and II dispersed in buffer, SIM_low_ and SIM^high^, whereas the droplet size measured after dispersion of SNEDDS III and IV seemed unaffected in all four media (Figure 5). At pH 1.2, the addition of cinnarizine had no effect on the droplet size of any of the SNEDDSs. Cinnarizine has a high solubility at pH 1.2, but a low solubility in the buffer at pH 6.5; at the former pH it has two positive charges and at the latter only one. Thus, more cinnarizine will distribute into the aqueous continuum at pH 1.2 and less will be dissolved inside the nanoemulsion droplets. As a result, cinnarizine takes up less space in the nanoemulsion droplets. This explanation has been confirmed by Pulsed Field Gradient NMR studies [44]. It was found that the diffusion coefficient of cinnarizine in a nanoemulsion in HCl resembled the diffusion coefficient of a solution of cinnarizine in HCl. The preference of cinnarizine for the nanoemulsion droplets is smaller for SNEDDS III and IV due to their higher content of hydrophilic excipients. 

Similar results regarding drug effect on droplet size increase have been reported in the literature; an increase in droplet size was observed with increasing drug loading for a microemulsion formulation containing nicardipine hydrochloride [45] and in another study, increased droplet size of nanoemulsions was observed when the poorly soluble compound halofantrine was incorporated, whereas no change was observed with another poorly soluble compound, probucol [41]. The increase in droplet size was only observed when the nanoemulsion containing halofantrine was dispersed in a media resembling SIM^high^ but not in saline. The difference in droplet size was only statistically significant for the nanoemulsion with the highest lipid content of the investigated nanoemulsions. Therefore, change in droplet sizes of nanoemulsions with incorporation of drug compound may depend on the dispersion media, the nature of the drug compound as well as on the nanoemulsion composition. The cause of the droplet size changes observed for cinnarizine incorporation in SNEDDS I and II, but not SNEDDS III and IV, is thus not clear. One explanation could be that more nanoemulsion droplets are available from SNEDDSs III and IV compared to I and II due to their smaller droplet size. As a result, fewer cinnarizine molecules are incorporated into each nanoemulsion droplet since the same number of cinnarizine molecules needs to get solubilized as the concentration of SNEDDS and cinnarizine is the same in the samples.

The droplet sizes of the SNEDDSs were determined in two different simulated intestinal media. One reflecting the fasted state SIM_low_ (5 mM bile acid) and one reflecting the fed state SIM^high^ (15 mM bile acid). These levels are in good agreement with the bile acid levels reported in man, ranging from 1.5 to 5.9 mM and 0.5 to 24 mM in the fasted and fed state, respectively [46]. When the SNEDDSs were dispersed in SIM at pH 6.5, a decrease in droplet size was observed compared with plain buffer at pH 6.5 (Figure 5). This decrease was observed both in the presence and absence of cinnarizine. Furthermore, the PDI values were increased as compared to SNEDDSs dispersed in buffer indicating a multimodal droplet size distribution (Table 6). However, we were not able to obtain two distinct droplet sizes probably because their size distributions are overlapped. The decrease in droplet size of the dispersed SNEDDSs in simulated intestinal media is most likely caused by the solubilization of oleic acid by the mixed bile acid/phosphatidylcholine micelles thereby reorganizing oleic acid from the nanoemulsion droplets resulting in a decrease in droplet size. In addition, solubilization of cinnarizine into the mixed bile acid/phosphatidylcholine micelles can displace cinnarizine from the nanoemulsion droplets and thereby decrease the size of the droplets. This explanation is further supported by the observation of a more pronounced decrease in particle size observed in SIM^high^ at the higher bile acid level (15 mM) compared to SIM_low_ (5 mM). This finding is apparently in contrast with a previous study where a difference in droplet size of nanoemulsions was not observed when dispersed in saline and in simulated intestinal media [41]. However, the nanoemulsions of the previous study contained a mixture of mono-, di-, and tri-glycerides as the lipid phase, which may be solubilized by mixed bile salt micelles to a lesser extent.

### 3.6. Cryo-TEM of SNEDDSs

**Figure 6:**
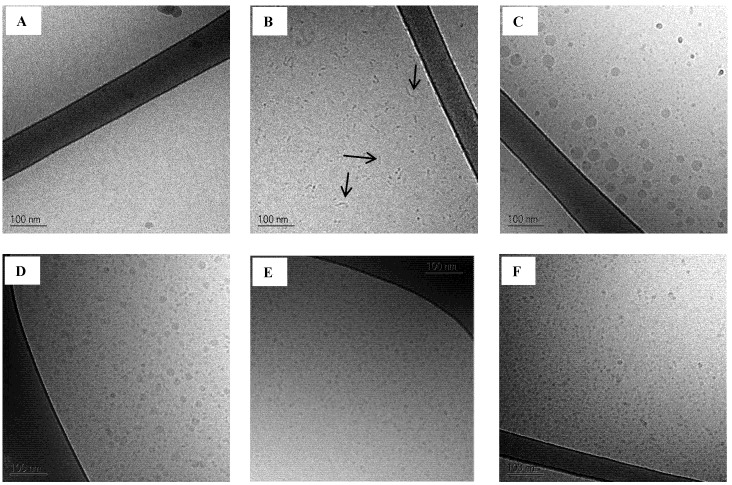
Cryo-TEM images of background (**A**: trizma maleate buffer pH 6.5 and **B**: SIM_low_ pH 6.5, arrows are pointing at the rod like structures) and SNEDDSs dispersed (1% *w*/*w*) in SIM_low_ pH 6.5;**C**: SNEDDS I;**D**: SNEDDS II;**E**: SNEDDS III and **F**: SNEDDS IV).

Cryo-TEM images of the four SNEDDSs dispersed in SIM_low_ (1% *w*/*w*) containing 5 mM sodium taurocholate and 1.25 mM phosphatidylcholine are shown in Figure 6C–F. Images of plain buffer and SIM_low_ are also shown as background reference (Figure 6A,B, respectively). SIM_low_ contains mixed taurocholate/phosphatidylcholine micelles and their rod like structure is evident on the image (Figure 6B). The finding of rod like structures of bile acid and phospholipid micelles is in accordance with previous studies [42,43].

When SNEDDSs are dispersed in SIM_low_ there is no evidence of rod-like structures. This finding further strengthens the theory that surfactants from the SNEDDSs interact with the mixed micelles in the intestinal simulated media. The Cryo-TEM images of the SNEDDSs in SIM_low_ show for SNEDDS I and II the coexistence of two droplet size populations (Figure 6C,D, respectively), whereas only one droplet size population is seen in SNEDDS III and IV (Figure 6E,F). The existence of two distinct droplet size distributions was expected from the PDI values obtained from DLS measurements (see above). (Table 6 and Table 7). It is not uncommon to overlook the presence of smaller droplets when bigger droplets are present when using DLS [47,48]. Two droplet size populations were probably seen due to the presence of more surfactant than necessary to cover the surface of the lipid content. As a result, surfactant aggregates with a distinct droplet size are also present in the nanoemulsions formed from SNEDDSs I and II. From SNEDDSs III and IV the low content/amount of lipid forms nanoemulsion droplets in the same range of the surfactant aggregates and therefore two distinct sizes are not observed.

**Table 7:** Droplet sizes of dispersed intact SNEDDS and SNEDDS composed of 50% or 100% mimicked digested sesame oil in SIM (1% (*w*/*w*). The SNEDDSs and the mimicked digested SNEDDSs contained 25 mg cinnarizine per gram. Measurements were performed at 37°C and the results are presented as the hydrodynamic diameter (nm)*^a^*^,*b*^.

SNEDDS	Media	Population 1		Population 2		Population 3		PDI
		Size (mean ± SD) *^c^*	% vol (range)	Size (mean ± SD) *^c^*	% vol (range)	Size (mean ± SD) *^c^*	% vol (range)	Mean ± SD
I	Intact in SIM_low_	36.4 ± 0.9 (10/10)	100	-	-	-	-	0.080 ± 0.022
	50% digested in SIM_low_	18.9 ± 1.3 (10/10)	99.2–100	668 ± 517 (5/10)	0.1–0.7	4721 ± 474 (3/10)	0.1	0.385 ± 0.168
	100% digested in SIM_low_	23.6 ± 1.4 (10/10)	97.6–98.6	313 ± 47 (10/10)	1.4–2.4	-	-	0.499 ± 0.057
	Intact in SIM^high^	32.4 ± 0.6 (9/9)	100	-	-	-	-	0.113 ± 0.010
	50% digested in SIM^high^	11.9 ± 2.3 (16/16)	17.2–100	3.4 (2/16)	14.4–82.8	71 ± 44 (3/16)	0.1–4.6	0.960 ± 0.139
	100% digested in SIM^high^	31.6 ± 4.7 (10/14)	11.7–100	2.8 (1/14)	74.1	10.2 ± 0.4 (8/14)	25.9–100	0.190 ± 0.012
II *^d^*	Intact in SIM_low_	23.3 ± 1.1 (10/10)	100	-	-			0.118 ± 0.023
	50% digested in SIM_low_	16.4 ± 1.0 (10/10)	99.4–100	655 (1/10)	<0.1	3387 ± 1560 (9/10)	<0.1–0.6	0.223 ± 0.031
	100% digested in SIM_low_	24.2 ± 2.6 (7/7)	98.6–99.3	605 ± 269 (7/7)	0.7–1.4			0.313 ± 0.007
	Intact in SIM^high^	18.1 ± 1.1 (10/10)	54.8–100	5.1 (2/10)	27.4–45.2			0.158 ± 0.007
	50% digested in SIM^high^	9.8 ± 0.4 (16/16)	100	-	-	-	-	0.205 ± 0.083
	100% digested in SIM^high^	9.1 ± 1.3 (10/10)	86.2–100	26.0 (2/10)	13.2–13.8	129 ± 7.8 (4/10)	0.1–0.2	0.283 ± 0.022
III	Intact in SIM_low_	13.9 ± 0.4 (10/10)	100			4954 (2/10)	<0.1	0.178 ± 0.034
	50% digested in SIM_low_	12.2 ± 0.3 (10/10)	99.9–100	581 ± 132 (3/10)	<0.1	4742 ± 649 (4/10)	<0.1	0.196 ± 0.055
	100% digested in SIM_low_	15.1 ± 2.3 (10/10)	60.2–100	6.7 (1/10)	39.8	2480 ± 2306 (8/10)	0.1–0.2	0.341 ± 0.047
	Intact in SIM^high^	10.4 ± 0.3 (10/10)	100	-	-			0.225 ± 0.038
	50% digested in SIM^high^	8.7 ± 0.4 (7/7)	100	-	-			0.184 ± 0.055
	100% digested in SIM^high^	7.8 ± 0.2 (16/16)	100	-	-	-	-	0.123 ± 0.045
IV	Intact in SIM_low_	11.4 ± 0.5 (10/10)	100	-	-	-	-	0.144 ± 0.031
	50% digested in SIM_low_	11.2 ± 0.2 (13/13)	100			5161 ± 91 (3/13	<0.1	0.218 ± 0.067
	100% digested in SIM_low_	10.5 ± 0.5 (13/13)	99.9–100	334 ± 183 (6/13)	<0.1–0.1	5025 ± 369 (4/13)	<0.1	0.231 ± 0.084
	Intact in SIM^high^	8.8 ± 0.2 (13/13)	100			-	-	0.117 ± 0.032
	50% digested in SIM^high^	8.2 ± 0.2 (16/16)	100	-	-	-	-	0.158 ± 0.058
	100% digested in SIM^high^	7.8 ± 0.1 (12/12)	100	-	-	-	-	0.147 ± 0.039

**Figure 7:**
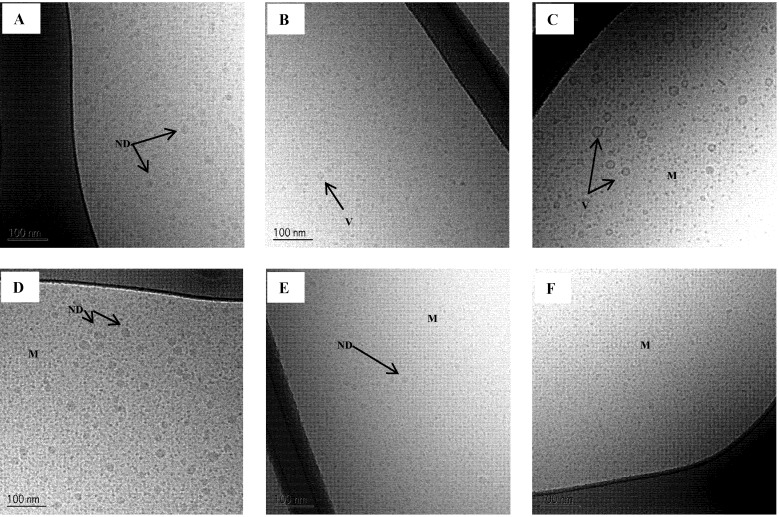
Cryo-TEM images of SNEDDS II intact and mimicked digested in intestinal simulated media. (**A**) Intact in SIM_low_;(**B**) 50% digested in SIM_low_ and (**C**) 100% digested in SIM_low_;(**D**) Intact in SIM^high^;(**E**) 50% digested in SIM^high^ and (**F**) 100% digested in SIM^high^. ND: Nanoemulsion droplet, M: Micelles, V: Vesicle.

*^a^* The droplet sizes of intact SNEDDSs in SIM are also shown in Figure 5. It is included here for comparative purposes; *^b^* All data from these measurements are included in this Table although droplet sizes that was only observed once out of 10 measurements and droplet sizes with a low volume fraction (<1%) may in some cases be considered artefacts; *^c^* The brackets define the number of times the particular droplet size appears / and the total number of measurements; *^d^* Cryo-TEM images of these samples are shown in Figure 7.

### 3.7. Triglyceride Composition in Sesame Oil

Knowledge of the composition of the sesame oil was required in order to mimic the composition of digested sesame oil. The fatty acid composition can vary with the origin of the oil [49] and, furthermore, variations between different batches of sesame oil can be anticipated since it is a natural product. Two batches of sesame oil were analyzed and the composition of the sesame oils is shown in Table 6. The fatty acids are designated as linoleic (L), oleic (O), palmitic (P), and stearic (S) acids. Combining these letters result in the different triglycerides, e.g., OOO is triolein. The composition of triglycerides in the two batches of sesame oil was practically identical; consequently, the results are shown together (Table 8). The analysis was run in triplicate on each batch and as Table 6 indicates, the method had a good repeatability. The chromatographic system was not able to distinguish between the triglycerides SOL and POO or between PSL and PPO. These compositions are therefore reported together. Since these triglycerides are only present in minor concentrations, it is not considered to have major impact on the results in the present study. 

**Table 8:** Composition of triglycerides in refined sesame oil. The fatty acids are designated as linoleic (L), oleic (O), palmitic (P), and stearic (S) acid.

Triglyceride	Compiled data *^a^*
	Mean ± SD ( *n* = 6)
LLL	11.1 ± 0.14
OLL	20.1 ± 0.06
PLL	7.9 ± 0.08
OOL	20.6 ± 0.13
POL	10.9 ± 0.05
PPL	1.6 ± 0.02
OOO	14.5 ± 0.09
SOL + POO	7.2 ± 0.04
PSL + PPO	1.1 ± 0.01
SOO	3.8 ± 0.04
SSL	1.2 ± 0.02

*^a^* Compiled data from two different batches of sesame oil (*n* = 3 for each batch).

### 3.8. Preparation of Mimicked Digested SNEDDSs

The composition of mimicked digested sesame oil was calculated from the content of triglycerides in the sesame oil. From these calculations, mimicked digested sesame oil was prepared by mixing the appropriate monoglycerides and fatty acids. The compositions are shown in Table 9. 

**Table 9:** Calculated composition of digested sesame oil and calculated composition of the mimicked digested sesame oil.

Component	Calculated composition of digested sesame oil (mg) *^a^*	Calculated composition of mimicked digested sesame oil (mg) *^b^*
Palmitic acid (C16)	75	75
Stearic acid (C18)	28	28
Oleic acid (C18–1)	249	248
Linoleic acid (C18–2)	285	285
Linolenic acid (C18–3)	-	3
MG-Palmitate	8	19
MG-Stearate	7	15
MG-Oleate	232	241
MG-Linoleate	159	171
MG-Linolenate	-	2
Diglycerides	-	12
Free glycerol	-	1
Sum	1043	1100

*^a^* The amount of digestion products (mg) formed from digestion of 1 g sesame oil; *^b^* The amount of digestion products (mg) in the mimicked digested sesame oil.

Table 9 shows that it was possible to closely mimic the composition of digested sesame oil. The main difference was the presence of a small amount of linolenic acid, monoglyceride-linoleate, diglycerides, and the presence of free glycerol in the mimicked composition. Diglycerides and free glycerol were present as impurities in the monoglyceride mixtures used. Generally, the mimicked composition contained higher amounts of monoglycerides. The sum of the masses of monoglycerides and fatty acids adds up to more than 1000 mg. Digestion of triglycerides adds two moles of water to the mass, but the mass of added water only accounts for approximately 4 mg. This difference may be explained by variability in the analysis of the sesame oil composition and round-off in the performed calculations

### 3.9. Droplet Sizes of Mimicked Digested SNEDDSs

Table 7 shows the droplet sizes of intact and mimicked digested SNEDDS dispersed in SIM_low_ and SIM^high^ with cinnarizine determined by DLS. The changes in droplet size induced by digestion vary between the different SNEDDSs. In general, cinnarizine did not affect the changes in droplet size observed as a function of digestion and therefore only the data from measurements of SNEDDSs containing cinnarizine is included in the paper. The droplet sizes of the intact and mimicked digested SNEDDS dispersed in SIM_low_ and SIM^high^ without cinnarizine are enclosed as supplementary material and is available electronically.

SNEDDS I and II dispersed in SIM_low_, displayed a larger droplet size compared to 50% and 100% mimicked digested SNEDDS, but going from 50% to 100% digestion the droplet size increased slightly. This was also seen for SNEDDS I in SIM^high^. In SIM^high^, the droplet size of SNEDDS II and III decreased when comparing intact to 50% mimicked digestion. However, further digestion (100%) did not change the droplet size. The droplet size of SNEDDS III in SIM_low_ had a tendency towards a decrease going from 0% to 50% mimicked digestion followed by a tendency to increase going from 50% to 100% mimicked digestion, similar to the observations for SNEDDS I and II. The droplet size in SNEDDS IV was not altered by digestion, probably due to its low content of the digestible excipient sesame oil.

In contrast to measurements made on intact SNEDDSs, measurements on mimicked digested SNEDDSs showed more than one droplet size population and the PDI values obtained from the measurements were generally high (Table 7). It was mainly larger droplet sizes that represent a small volume fraction (%). These larger droplets do not appear in all measurements; however, they indicate changes in the nanoemulsion systems and formation of larger colloidal structures as a function of digestion. From Table 7 it is seen that SNEDDS III and IV, containing the highest surfactant levels, do not form larger colloidal structures as a function of digestion when dispersed in SIM^high^. This shows that the high level of taurocholic acid together with the surfactants of the formulation is able to solubilize the digestion products without the formation of larger colloidal structures. The low level of taurocholic acid is not sufficient to prevent the formation of larger colloidal structures from digested SNEDDSs III and IV.

### 3.10. Cryo-TEM of Mimicked Digested SNEDDS

Cryo-TEM images of intact and mimicked digested SNEDDS II dispersed in SIM_low_ and SIM^high^ are shown in Figure 7. The results are in accordance with the result from the DLS measurements. In SIM_low_ a reduction in droplet size is seen at 50% digestion and then an increase in droplet size is observed at 100% digestion. From the Cryo-TEM images it is evident that the larger structures at 100% digestion are mainly vesicles. From DLS measurements, it is not possible to distinguish between oil droplets or vesicles since it is the hydrodynamic size of the colloids that is determined by this technique. Therefore, the use of an imaging technique to supplement DLS measurements is preferable. At 50% digestion, a few vesicles were also observed in SIM_low_. 

**Figure 8:**
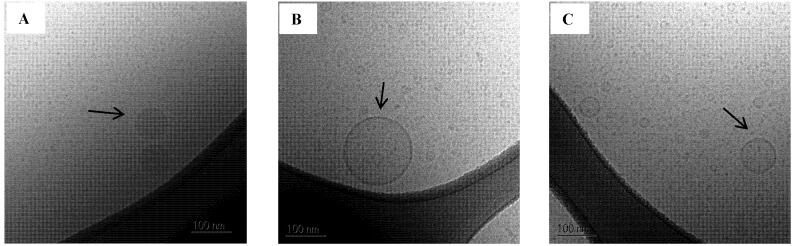
Cryo-TEM images of SNEDDS II 100% mimicked digested in SIM_low_. (**A**) Oil droplets;(**B**) and (**C**) Vesicles. The scale bar is 100 nm.

A few larger oil droplets and vesicles approx. 80–250 nm in diameter were also observed in the 100% mimicked digested sample in SIM_low_ (Figure 8). Larger colloidal structures were also observed by DLS, however these appeared to be somewhat bigger than what was seen on the Cryo-TEM images. In contrast, vesicles were not observed in SIM^high^ when SNEDDS II was digested. After 50% mimicked digestion the droplet size decreased compared to the intact SNEDDS and was unchanged comparing 50% to 100% mimicked digestion. The absence of vesicles when mimicked digested SNEDDS II was dispersed in SIM^high^ is caused by the higher concentration of bile acid as compared to the SIM_low_ media. In SIM^high^ there is sufficient surfactant to keep the lipolysis products solubilized in smaller colloidal structures whereas in SIM_low_ the amount of surfactant is not sufficient to prevent the formation of larger colloidal structures e.g., vesicles and oil droplets.

The results obtained here substantiate previous results by Fatouros *et al.* 2009 using a dynamic *in vitro* lipolysis model and Cryo-TEM imaging looking at the morphology during digestion of a SNEDDS in simulated fed and fasted state [50]. It was found that the simulated fed state resulted in the formation of micelles whereas in the fasted state a mixture of oil droplets, vesicles and micelles was formed [50]. The extent of digestion was 40% and 50% in the fed and fasted state, respectively. To further validate our approach of making mimicked digested SNEDDSs for mechanistic studies Cryo-TEM images of actually digested SNEDDS from the present study would be needed.

In the present study, the results are snap shots of the digestion of SNEDDSs, however the absorption of digestion products was not taken into account. The absorption of lipolysis products may lead to a higher bile acid to lipolysis products ratio, which may favor micelle formation over vesicle formation. It is therefore anticipated that the rate of digestion of SNEDDSs and the rate of absorption of the digestion products may influence the colloidal systems that the drug compound is absorbed from. For some compounds it has been shown that the nano-structural composition of the media and not just the total surfactant contents has an impact on the solubilization capacity of the specific medium [51] and that the nano-structural composition can potentially impact the absorption of compounds from such media. For SNEDDS I and II the level of bile salt have a greater impact on the droplet sizes obtained after mimicked digestion than SNEDDS III and IV. This may implicate that the performance of some SNEDDSs may be more dependent on the bile salt level in the intestinal fluids than others. Significant differences in bioavailability between the four SNEDDSs have been obtained [44]. SNEDDS I and II resulted in lower bioavailability when compared to SNEDDS III and IV. However, exactly what causes the differences *in vivo* is not clear cut. Data from studies like the present aids in obtaining a better understanding of SNEDDS formulations and linked to *in vivo* data more can be learned on factors important for increasing the bioavailability of poorly water-soluble compounds from SNEDDSs.

## 4. Conclusion

In the present study, four SNEDDSs with well-defined compositions were developed and characterized. The formulations were developed in a way that made it possible to create mimicked digested SNEDDSs in the laboratory without the use of *in vitro* lipolysis models and lipases. The developed SNEDDSs differed in composition, droplet sizes, dispersion time, as well as the solubility of cinnarizine in their preconcentrates. Droplet size characterisation by DLS showed that biorelevant media containing bile acid and phosphatidylcholine decreased the size of the nanoemulsion droplets compared to dispersion in plain buffer. The nanoemulsion droplets formed from the SNEDDSs with the highest lipid content increased in size when they contained cinnarizine, whereas the systems with less lipid was unchanged by the presence of cinnarizine. However, dispersion in acidic environment (pH 1.2) removed the effect that cinnarizine had on size, likely due to a higher solubility of the drug in the aqueous continuum. In the present study, smaller micelles were observed on Cryo-TEM images that were not detected by DLS in samples of SNEDDSs dispersed in biorelevant media. By Cryo-TEM the morphology of one of the SNEDDSs as a function of the extent of digestion was studied. First, a decrease of droplet size going from 0% to 50% digestion and hereafter an increase in size after 100% digestion was observed. Depending on the biorelevant medium used for dispersion either vesicles or micelles were formed at 100% digestion; a low content of bile acid resulted in vesicles and a high content lead to the formation of micelles. In summary, we have developed SNEDDSs suited for performing mechanism-based studies on the solubilization and absorption of poorly water-soluble drug compounds. This study highlighted the interplay between drug, formulation and environment, e.g., the effects of pH, SIM and digestion using cinnarizine as a model drug compound. Additional work utilizing these SNEDDSs may be used to unravel the effect of digestion on the absorption from SNEDDSs.

## Supplementary Files

Supplementary File 1 Supplementary Information (PDF, 75 KB)
