# Supplementary material for: SNEDDS Containing Poorly Water Soluble Cinnarizine; Development and in Vitro Characterization of Dispersion, Digestion and Solubilization
Source: Pharmaceutics. 2012 Dec 14;4(4):641–65. doi: 10.3390/pharmaceutics4040641 (PMC3834928; doi:10.3390/pharmaceutics4040641)
Supplement: Supplementary File 1 — Supplementary Information (PDF, 75 KB) [file pharmaceutics-04-00641-s001.pdf]

Supplementary Information

Table S1. Droplet sizes of dispersed intact SNEDDS and SNEDDS composed of 50% or 100% digested sesame oil in SIM (1%, *w/w*). Measurements were performed at 37 °C and the results are presented as the hydrodynamic diameter (nm) [a,b].

SNEDDS	Media	Population 1		Population 2		Population 3		PDI
		Size (mean ± SD) [c]	% vol (range)	Size (mean ± SD) [c]	% vol (range)	Size (mean ± SD) [c]	% vol (range)	Mean ± SD
I	Intact in SIM$_{low}$	30.0 ± 0.7 (10/10)	100	-	-	-	-	0.097 ± 0.018
	50% digested in SIM$_{low}$	18.7 ± 0.6 (10/10)	99.4–99.9	693 ± 627 (6/10)	0.1–0.6	4298 ± 703 (7/10)	0.1–0.2	0.297 ± 0.032
	100% digested in SIM$_{low}$	27.7 ± 1.4 (10/10)	78.0–94.9	444 ± 134 (10/10)	4.4–8.5	3294 ± 1055 (9/10)	0.3–17.5	0.565 ± 0.023
	Intact in SIM^{high}	26.3 ± 1.1 (10/10)	100	-	-	-	-	0.147 ± 0.016
	50% digested in SIM^{high}	10.8 ± 2.6 (16/16)	99.3–100	194 ± 259 (13/16)	0.2–0.6	2905 ± 985 (4/16)	0.2–0.6	0.385 ± 0.172
	100% digested in SIM^{high}	21.9 ± 6.7 (14/14)	23.5–100	7.9 ± 1.6 (5/14)	58.5–80.6	3227 ± 1241(11/14)	0.1–0.7	0.258 ± 0.008
II	Intact in SIM$_{low}$	19.5 ± 0.7 (10/10)	99.9–100	-		4424 ± 159 (3/10)	0.1	139 ± 0.058
	50% digested in SIM$_{low}$	18.0 ± 1.7 (10/10)	98.7–99.8	444 ± 133 (10/10)	0.2–1.3	-	-	0.349 ± 0.033
	100% digested in SIM$_{low}$	27.4 ± 0.4 (10/10)	74.9–97.5	381 ± 64 (10/10)	2.5–3.6	10.3 (1/10)	22.5	0.496 ± 0.018
	Intact in SIM^{high}	15.2 ± 0.7 (10/10)	100	-	-	-	-	0.158 ± 0.006
	50% digested in SIM^{high}	9.5 ± 0.2 (10/10)	100	-	-	4273 ± 192 (3/10)	<0.1	0.208 ± 0.057
	100% digested in SIM^{high}	9.7 ± 1.3 (10/10)	79.8–100	29.4 ± 1.4 (3/10)	15.9–20.1	3897 ± 821 (8/10)	<0.1–0.1	0.319 ± 0.047
III	Intact in SIM$_{low}$	13.0 ± 0.3 (13/13)	99.9–100	243 ± 132 (6/13)	<0.1–0.1	5075 ± 247 (4/13)	<0.1	0.223 ± 0.089
	50% digested in SIM$_{low}$	12.0 ± 0.5 (13/13)	99.8–99.9	406 ± 359 (13/13)	<0.1–0.2	4926 ± 339 (4/13)	<0.1	0.362 ± 0.089
	100% digested in SIM$_{low}$	16.8 ± 3.8 (14/14)	38.2–99.6	481 ± 175 (14/14)	0.2–0.8	6.4 (2/14)	46.1–61.5	0.406 ± 0.011
	Intact in SIM^{high}	10.2 ± 0.3 (10/10)	100	-	-	-	-	0.140 ± 0.049
	50% digested in SIM^{high}	8.3 ± 0.1 (13/13)	100	-	-	-	-	0.128 ± 0.054
	100% digested in SIM^{high}	7.5 ± 0.2 (16/16)	100	-	-	-	-	0.152 ± 0.049
IV	Intact in SIM$_{low}$	11.2 ± 0.4 (10/10)	100	-	-	-	-	0.175 ± 0.040
	50% digested in SIM$_{low}$	10.8 ± 0.2 (10/10)	100	243 ± 51.6 (3/10)	<0.1	5217 (2/10)	<0.1	0.267 ± 0.085
	100% digested in SIM$_{low}$	10.4 ± 0.6 (10/10)	99.9–100	188 ± 24.9 (6/10)	<0.1–0.1	-	-	0.346 ± 0.070
	Intact in SIM^{high}	8.7 ± 0.3 (10/10)	100	-	-	-	-	0.168 ± 0.015
	50% digested in SIM^{high}	7.9 ± 0.3 (13/13)	100	-	-	-	-	0.151 ± 0.059
	100% digested in SIM^{high}	7.6 ± 0.2 (13/13)	100	-	-	-	-	0.178 ± 0.045

[a] The droplet sizes of intact SNEDDSs in SIM are also shown in Figure 5. It is included here for comparative purposes; [b] All data from these measurements are included in this table although droplet sizes that was only observed once out of 10 measurements and droplet sizes with a low volume fraction (<1%) may in some cases be considered artefacts; [c] The brackets define the number of times the particular droplet size appears/and the total number of measurements.
